# *In silico* Design of a Multivalent Vaccine Against *Candida albicans*

**DOI:** 10.1038/s41598-020-57906-x

**Published:** 2020-01-23

**Authors:** Shikha Tarang, Varun Kesherwani, Blake LaTendresse, Laramie Lindgren, Sonia M. Rocha-Sanchez, Michael D. Weston

**Affiliations:** 1 Creighton University School of Dentistry, Department of Oral Biology, Omaha, NE 68178 USA; 2 Child Health Research Institute, University of Nebraska Medical Center, Omaha, NE 68198 USA

**Keywords:** Protein analysis, Peptide vaccines

## Abstract

Invasive candidiasis (IC) is the most common nosocomial infection and a leading cause of mycoses-related deaths. High-systemic toxicity and emergence of antifungal-resistant species warrant the development of newer preventive approaches against IC. Here, we have adopted an immunotherapeutic peptide vaccine-based approach, to enhance the body’s immune response against invasive candida infections. Using computational tools, we screened the entire candida proteome (6030 proteins) and identified the most immunodominant HLA class I, HLA class II and B- cell epitopes. By further immunoinformatic analyses for enhanced vaccine efficacy, we selected the 18- most promising epitopes, which were joined together using molecular linkers to create a multivalent recombinant protein against *Candida albicans* (mvPC). To increase mvPC’s immunogenicity, we added a synthetic adjuvant (RS09) to the mvPC design. The selected mvPC epitopes are homologous against all currently available annotated reference sequences of 22 *C. albicans* strains, thus offering a higher coverage and greater protective response. A major advantage of the current vaccine approach is mvPC’s multivalent nature (recognizing multiple-epitopes), which is likely to provide enhanced protection against complex candida antigens. Here, we describe the computational analyses leading to mvPC design.

## Introduction

Invasive candidiasis (IC) is one of the most common public health problems and is a major therapeutic challenge^1^. Increasing frequency of the pre-disposing risk factors has led to a remarkable 20-fold higher incidence of IC in just two decades^2^. However, unlike other fungal infections, IC is usually due to an endogenous candida overgrowth at mucosal surfaces (mucosal candidiasis)^3^. *Candida albicans (C. albicans)* is the most common etiological agent of IC and is found in ~ 60% of clinical isolates of candidiasis^4^. In healthy individuals, *C. albicans* co-exists with the host in a harmless commensal (yeast) form without causing disease^5^. However, certain underlying conditions (e.g., major surgery, broad-spectrum antibacterial therapy, immunodeficiency diseases [AIDS, diabetes, cancer chemotherapy, etc.], or even premature birth) can cause candida to become pathogenic^6^. The pathogenesis of candida requires a switch from its commensal yeast form to a pathogenic fungal (hyphal) form^5^. The presence of hyphae enables candida to cause widespread damage to the underlying tissues. If the infection remains unchecked, candida can gain access to the host vasculature^5^. Once candida enters the bloodstream, it can spread throughout the body, causing life-threatening IC with a high mortality rate of 60%^7^. The current treatment with a limited set of available drugs leads to high drug-induced systemic toxicity and is associated with an increased emergence of anti-fungal-resistant candida species^2^. Therefore, despite the progress in medicine in general, IC is a leading cause of mycoses-related deaths^8^.

Immunosuppression is the leading cause of fatal, invasive candida infections. In healthy individuals, the ability of candida to alter its morphology is largely kept in check by an effective immune response^9^. The significance of an effective immune response in preventing IC, suggests that mimicking a natural immune response to candida may be an effective strategy to control its burden. In line with this, vaccination approaches against IC include PEV7^10^ and NDV3^11^, which have completed Phase I studies and are in further stages of immunogenicity and toxicity testing. PEV7 is a virosomal vaccine to protect women suffering from chronic vaginal yeast infections (vulvovaginal candidiasis or VVC). It consists of a truncated recombinant secreted aspartic protease 2 (Sap2), currently in clinical testing by Pevion BiotechAG^10^. NDV3 targets the recombinant N-terminal region of the hyphal protein agglutinin-like sequence three protein (rAls3p-N) and is being developed by NovaDigm Therapeutics^12^. Another vaccination approach from Novartis Pharmaceuticals (Efungumab) based on monoclonal antibody targeting the heat shock protein 90 (Hsp90) progressed through to a Phase III clinical trial but was abandoned in later stages of development due to safety concerns^13^. Finally, a prophylactic and therapeutic IgM-monoclonal antibody (MAb B6.1) by LigoCyte Pharmaceuticals that targeted (1 → 2)-β-mannotriose also failed during development^14^. Thus, there are no FDA-approved candida vaccines for human use.

The failure of current vaccination approaches in eliciting an effective anti-candida immune response is attributed to a variety of reasons. One of the major drawbacks is candida’s ability to evolve and ultimately escape the host immune surveillance^15^. Therefore, simultaneous targeting against multiple candida epitopes (multivalence) is expected to provide improved outcomes. So far, the multivalent-vaccine approach has not been adopted against *C. albicans*. Due to the complex nature of candida antigens and its ability to escape host-immune surveillance, a multi-epitope vaccine will likely be more beneficial in inducing a stronger and broader immune responses^15,16^. While most vaccines (to date) focused only on one antigen, our approach involves the simultaneous targeting of multiple candida antigens which are molecularly linked to form a single recombinant protein. Our strategy involves experimental validation of single peptide antigens whereby positive peptide epitopes can be combined to design a multivalent recombinant protein vaccine against *C. albicans*. The findings presented in the current study detail *in silico* epitope mapping and provide future directions for vaccine design against *C. albicans*.

## Methods

### Antigenicity prediction

*C. albicans* sc5314 (the most common clinical isolate)^17^ was used as a reference strain to retrieve its entire proteomic sequences (consisting of 6030 proteins) from the NCBI protein database. Next, each of these proteins fasta sequences was run on VaxiJen server, which utilizes an alignment-independent method based on principal amino acid properties^18^. Based on the published literature^19^, an antigenicity probability >0.9 was considered acceptable for subunit vaccines. The proteins were filtered based on their antigenicity score (>0.9) and subcellular localization (extracellular, plasma membrane or nuclear), using a web server (CELLO2GO^20^) for protein subcellular localization prediction, which shortlisted the number of proteins to 36 (Supplementary file 1, yellow and green highlight). Since our goal is to stop candida’s switch to the pathogenic fungal form (without affecting its commensalism), we selected five of the 36 proteins (Als4p, Als3p, Fav2p, Als2p, Eap1p) (Supplementary file 1, green highlight) with known functions in hyphae formation. Further, we included three more hyphal proteins (Hyr1p, Hwp1p, Sap2p) (Supplementary file 1, blue highlight) from published studies^21^. The antigenicity score of these proteins was slightly below our cutoff (0.9), but due to their role in candida hyphae formation, we selected them for further development **(**Table 1**)**.

**Table 1:** Most-antigenic proteins in *C. albicans* proteome.

Accession Number	Vaxijen Analyses	Subcellular Localization
XP_710425.2	Als4p [Candida albicans SC5314] Overall Antigen Prediction = 1.2231 (Probable ANTIGEN)	*Exc/PM/Nu
XP_710435.2	Als3p [Candida albicans SC5314] Overall Antigen Prediction = 0.9490 (Probable ANTIGEN)	Exc/PM/Nu
XP_711172.1	Fav2p [Candida albicans SC5314] Overall Antigen Prediction = 0.9661 (Probable ANTIGEN)	Exc/Nu
XP_712646.2	Als2p [Candida albicans SC5314] Overall Antigen Prediction = 1.2323 (Probable ANTIGEN)	Exc/PM/Nu
XP_714572.2	Eap1p [Candida albicans SC5314] Overall Antigen Prediction = 1.0124 (Probable ANTIGEN)	Exc/Nu
XP_722183.2	Hyr1p [Candida albicans SC5314] Overall Antigen Prediction = 0.8286 (Probable ANTIGEN)	PM
XP_709961.2	Hwp1p [Candida albicans SC5314] Overall Antigen Prediction = 0.7474 (Probable ANTIGEN)	PM
XP_711047.1	Sap2p [Candida albicans SC5314] Overall Antigen Prediction = 0.7035 (Probable ANTIGEN)	Exc

*Extracellular/Plasma membrane/Nuclear.

### Epitope mapping

Using a NetCTL server^22^, we screened for the most antigenic HLA class I epitopes. HLA class I alleles are sub-grouped into 12 superfamilies (A1, A2, A3, A24, A26, B7, B8, B27, B39, B44, B58, B62). We screened each of the eight-hyphal proteins against each of the HLA class I superfamily (a total of 8 × 12 = 96 queries). The threshold values used were (HLAI binding [epitope identification] >0.75, weight on proteasomal C- terminal cleavage = 0.15; and weight on TAP [transport efficiency] = 0.05)^22^ (data not shown). For HLA class II epitope binding, we used the IEDB prediction server^23^, with low percentile rank and IC_50_ value as the selection criteria. Based on the spatial structure of the epitopes, the B-cell epitope structure can be categorized as continuous (linear) and discontinuous (conformational) epitopes^24^. For identification of linear B-cell epitopes, we used the BCPreds server^25^, while discontinuous B-cell epitope analyses were done using the IEDB prediction server^23^. B-cell epitopes were 20mer in length. Supplementary file 2 lists 240 selected HLA class I, HLA class II and B-cell epitopes in each of the 8 selected hyphal proteins. Next, to eliminate the possibility of *overlapping epitopes* we ran the 240 selected epitopes on the IEDB cluster analysis algorithm and filtered the epitopes for sequence identity (Supplementary file 2). This reduced the number of epitopes to a total of 214. Binding predictions for 10 conserved HLA class II T- cell epitopes (Table 2) and phenotype frequency of HLA allele type was analyzed using IEDB prediction server. IEDB recommended consensus percentile rank of the top 10% was taken for making selections (Supplementary file 3).

### Conservation analyses

Further, we did the conservation analysis of 214 epitopes with 22 *C. albicans* strains with an annotated sequence available on NCBI. The selection of epitopes in the conserved regions of the *C. albicans* sequence will ensure higher coverage and protection against candidiasis. Using this analysis tool, we found 18 epitopes showing a 100% conservancy (Table 2). Vaccine designing was done using these 18 epitopes.

**Table 2:** Selected HLA class I (9mer), HLA class II (15mer) and B- cell (20mer) epitopes.

EPITOPE	PROTEIN
SNGLNDWNYPISSES	XP_710425.2 Als4p
LVTYQNVPAGYRPFV	XP_710425.2 Als4p
MLLQFLLLSLCVSVA	XP_712646.2 Als2p
RILLSRILPSLSQAV	XP_710425.2 Als4p
MLQQYTLLLIYLSVA	XP_710435.2 Als3p
PSLNKVSTLFVAPQC	XP_710435.2 Als3p
HVGITKGLNDWNYPV	XP_710435.2 Als3p
MFLKNIFIALAIALL	XP_711047.1 Sap2p, XP_718053.2 Sap1p, XP_723210.1 Sap3p
NNMRLTFGAAIIGIA	XP_709961.2 Hwp1p
ISTFEGAGNNMRLTF	XP_709961.2 Hwp1p
PYDKCQLLF	XP_711047.1 Sap2p
TVTAPPGGTDTVIIREPPNP	XP_710425.2 Als4p, XP_712646.2 Als2p, XP_718077.1 Als1p
KTNEAGGSYDNVPVTLKKQG	XP_711047.1 Sap2p
NSPDAATGQIIFGGVDNAKY	XP_711047.1 Sap2p
VVKTPKAFPVTNGQEGKTSK	XP_711047.1 Sap2p
EFAASLQGDDGQPYDKCQLL	XP_711047.1 Sap2p
ADFCKQKGTYDPSGSSASQD	XP_711047.1 Sap2p
LDGSASTTATVTPSLTDLQA	XP_711172.1 Fav2p

### Peptide fusion

**Figure 1:**
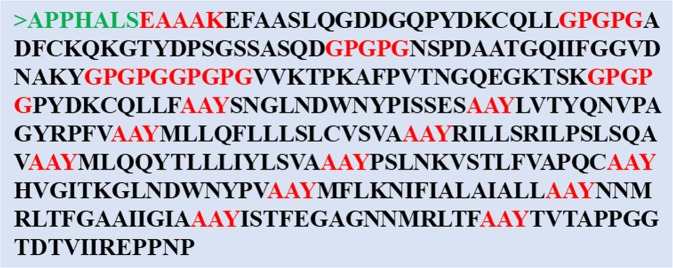
mvPC design showing peptide fusion by molecular linkers (red) and synthetic RS09 adjuvant (green).

**Figure 2:**
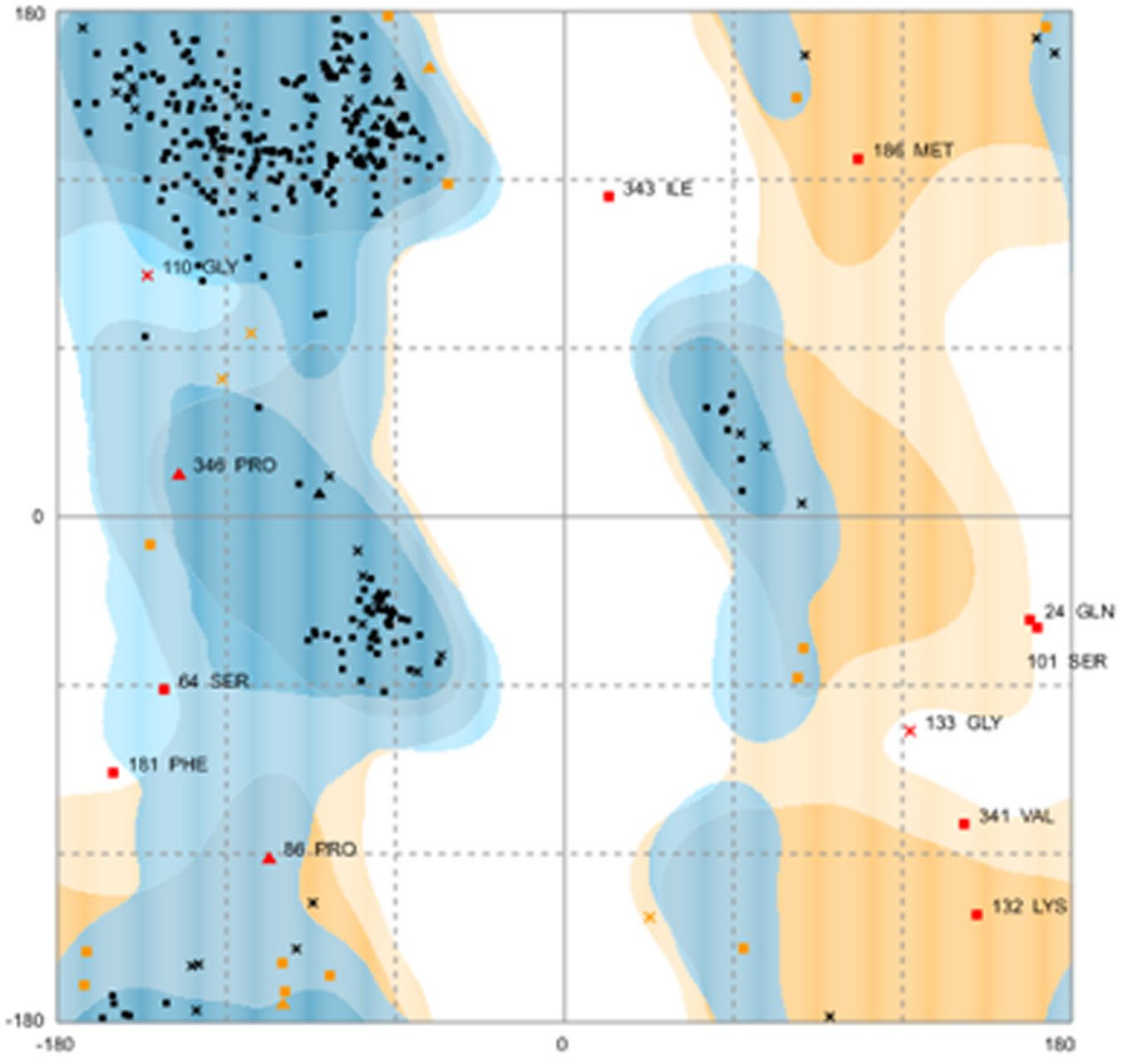
Ramachandran plot showing mvPC stability - Number of Residues in favored region 317 (91.4%); allowed region 18 (5.2%); and outlier region 12 (3.5%).

To ease any potential issues with the delivery of 18 single peptides, we constructed a fusion protein using molecular linker peptides. For example, intra Tc and Th epitopes were joined by AAY and GPGPG linker, respectively **(**Fig. 1). We also incorporated a TLR4 agonist RS09 (APPHALS) at the N- terminal end of the final vaccine construct. Molecular linking of 18- single candida epitopes will facilitate better *in vivo* antigen processing and presentation. The choice of linker peptides and the adjuvant was based on the study published by Pandey *et al*.^19^. RS09 mimics lipopolysaccharide (LPS), a natural TLR4 ligand^26^. Thus, the presence of RS09 enables co-stimulation of TCRs, driving a more robust immune activation. Use of synthetic adjuvants (RS09) is a safer approach and considered an advancement over traditional vaccination approaches, such as using Freund’s adjuvant^27^. The Tc epitope and adjuvant were joined by the EAAAK linker sequences **(**Fig. 1**)**. Next, we performed the secondary structure analyses of the final mvPC vaccine construct using the RaptorX server^28,29^. Our initial analyses showed ~10% protein disorder. To further enhance protein stability, we identified the region of disorders and removed the peptide (GPGPGKTNEAGGSYDNVPVTLKKQG) (not shown in Fig. 1). Further rearrangements led to a final 349aa-long mvPC vaccine with 1% protein disorder **(**Fig. 2).

### Sequence validation

Since fusing several epitopes can change the 3D- spatial arrangement of epitopes, we decided to validate T- cell (HLA class I and HLA class II) and B- cell (linear and discontinuous epitopes) in the final protein sequence. We found 100% conservation of 9mer (HLA class I), 15mer (HLA class II) and 20mer (B-cell) epitopes. We also found that the selected epitopes show 80–95% conservancy with other candida species (*C. dubliniensis, C. parapsilosis*, and *C. orthopsilosis)* (data not shown).

### Biophysiochemical analyses

The biophysiochemical characteristics of the final mvPC vaccine were evaluated on the ProtParam server^30,31^. A predicted molecular weight of 36.3 kDa favored the antigenicity of the vaccine construct. mvPC’s isoelectric point (pI) is 6.14, suggesting that it is near neutral pH. The estimated half-life in mammalian reticulocytes is predicted to be ~4.4 h. The instability index (II) of the final mvPC vaccine is computed to be 33.79. Based on these analyses, mvPC vaccine is stable.

### Antigenicity and allergenicity analyses

The efficacy of any vaccine is primarily determined by its antigenicity (i.e., potential to trigger an immune response). The antigenicity of the final mvPC vaccine was tested using ANTIGENpro^32^ and VaxiJen^33,34^ and was found to be 0.87 and 0.80, respectively. These values of antigenicity of the final mvPC vaccine are acceptable and comparable to other published subunit vaccines^19^. Elicitation of an allergic response will be unacceptable to vaccine administration; therefore, to rule out any potential allergic response, allergenicity was tested using the AllerTOP^35^ server, and the mvPC vaccine was found to be non-allergic. We also performed proteasome cleavage analysis of final mvPC construct using NetChop3.1 and MHCII-NP on IEDB server. The final mvPC was cleaved by proteasomes to generate the predicted T- cell epitopes identified in Table 2 (Supplementary file 4).

### mvPC tertiary structure analyses

To visualize the tertiary structure of the final mvPC vaccine, we used a template-based tertiary structure prediction algorithm, RaptorX^28,29^. Given the input sequence, RaptorX predicts its secondary and tertiary structures, as well as solvent accessibility and disordered regions. Using RaptorX, mvPC was found to be a single-domain stable protein. RaptorX used 2y7lA (top-ranked template) for mvPC structure prediction. A p-value of 5.67e-10 confirms the confidence in the mvPC tertiary structure prediction. Overall uGDT was found to be 106 (uGDT >50 is considered a good prediction). In the final structure, 7 (2%) positions were predicted to be disordered. Secondary structures in protein are 6%H, 40%E, and 53%C, while solvent access probability is 29%E, 39%M, and 30%B.

**Figure 3:**
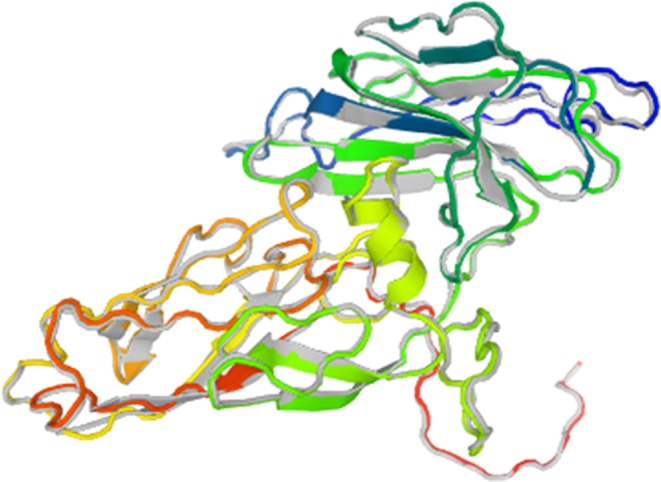
mvPC 3D- structure generated using Galaxy refine server.

The Rampage server was used to identify the tertiary structure stability prediction of the final mvPC construct. The number of residues in the favored region was 310 (89.3%); the number of residues in the allowed region was 19 (5.5%); and the number of residues in the outlier region was 18 (5.2%) (data not shown). We further refined the final 3D- structure using the Galaxy refine server and found improvement in the favored region. The number of residues in the favored region was 317 (91.4%); the number of residues in the allowed region was 18 (5.2%); and the number of residues in the outlier region was 12 (3.5%) **(**Fig. 2**)**. We finally decided to work with the refined model and generated a 3D- structure using Galaxy refine server **(**Fig. 3). We performed aggregation analysis of unrefined 3D- structure of mvPC using Aggrescan3.0 in dynamic mode. The average A3D score of input structure is 0.036 and minimum energy of a model_7 in dynamic mode is −0.0742 (Supplementary file 4).

## Results and Discussion

High drug-toxicity and emergence of drug-resistant candida species has led to a wide-consensus on the development of immunotherapeutic approaches against IC^36–39^. However, the development of an effective vaccine molecule using conventional approaches involves inoculation of live-attenuated or inactivated pathogen, requiring laborious biochemical, immunological, and microbiological methods to identify the antigenic components^40^. Moreover, these approaches are limited in application. For example, a large number of patients with immunosuppressive conditions (such as cancer, solid organ or hematopoietic stem cell transplant, HIV, acquired or congenital immunodeficiency)^6,41^ are at a higher-risk for systemic candida infection and require alternate approaches to immunization.

Recent advances in genomic and proteomic approaches have revealed the power of computational tools to design effective and safe new-generation vaccines^42,43^. The success of immunoinformatic approaches in vaccinology was first demonstrated in 2013 with the licensing of Bexsero and Trumenba, protein-based vaccines against serotype B meningococcal vaccines^44,45^. Since then, these approaches have been used successfully in the design of subunit vaccines against some of the most infectious and difficult pathogens, including, *Streptococcus pneumoniae* (pneumonia)^46^, *Escherichia coli*^47^*, Clostridium difficile*^48^*, Chlamydia trachomatis*^49^, human cytomegalovirus (HCMV), respiratory syncytial virus (RSV), HIV, influenza and dengue viruses^50^, which demonstrate the significance of immunoinformatic approaches in facilitating the vaccine development process.

The present study represents the first proteome-wide immunoinformatic approach to identify the immunodominant epitopes and design a multivalent subunit vaccine against *C. albicans*. Using web-based servers, we screened the entire candida proteome (consisting of 6030 proteins), to identify the most immunodominant candida antigens. Of note, our immunogenicity analyses focused on identifying epitopes in the hyphal proteins, which help candida adhere to and invade epithelial cells, resulting in severe damage to the host cells^5^. Thus, targeting hyphae will preserve candida’s yeast form without affecting host-fungal commensalism. Eight antigenic proteins with known functions in hyphal formation (Als4p, Als3p, Fav2p, Als2p, Eap1p, Hyr1p, Hwp1p, Sap2p) were selected further for epitope mapping (Table 1). Immunogenicity testing led to the selection of 18 unique epitopes [10 CD4^+^ T- helper or Th (15mer), 7 B- cell (20mer) and 1 CD8^+^ T- cytotoxic or Tc (9mer)]. The rationale for selecting these epitopes lies in the significance of Th cells in recognizing HLA class II proteins and in turn activating both B- cells to secrete antibodies and activating Tc cells to kill infected target cells^51^. Antibody response to *C. albicans* is ideal to target carbohydrate moieties on the fungal cell wall, as well as some secreted proteins (secreted aspartyl proteinase or SAP^52^) and has been shown to be effective in providing resistance against IC^53^. Although Tc play a minor role in natural immunological defense against candida and have not been studied much, previous studies show that Tc are effective in controlling fungal infection post-vaccination^54–56^. Therefore, a combination of strong humoral and cell-mediated immune responses is likely to confer an effective immune response against pathogenic candida. Conservancy analysis also show that the selected epitopes (in addition to the eight hyphal proteins), are also present in other candida proteins showing sequence homology (Sap1p, Sap 3p and Als1p, Table 2). Further, while designing the mvPC vaccine we have not only identified immunodominant epitopes in the antigenic proteins of the reference strain but also checked their conservancy across all known 22 strains of *C. albicans* whose proteome sequences are available in NCBI. Our selected antigens are in the highly-conserved regions of the candida proteome, offering protection against the 22 currently known candida strains. Selection of conserved candida epitopes also minimizes the chances of mutation in all the conserved regions simultaneously, limiting the emergence of new-resistant candida species. The selected epitopes also show conservation (a.k.a immunoprotection) with other pathogenic candida species (*C. dubliniensis, C. parapsilosis*, and *C. orthopsilosis*). Lastly, selected epitopes show HLA binding affinity in all 27 reference alleles of HLA class II with a number of epitopes demonstrating binding ability with multiple HLA subtypes. The presence of RS09 (via activation of the TLR4 signalling pathway) is expected to boost anti-candida immune response by skewing Th response towards the Th17 subtype which plays a major role in mounting an immune response in clearance of pathogenic *C. albicans*^57,58^. RS09 has previously been shown to provide better adjuvanticity with fewer side effects in HIV-1^59^.

It is important to note here that the efficacy of peptide vaccine is also largely dependent on the HLA type of the individual. A functional response will only be generated in individuals with a particular HLA type capable of binding a particular peptide epitope. Studies are underway to assess immunogenicity using single peptides in HLA-matched PBMC samples. Experimental testing of individual single peptides prior to linking them in the recombinant mvPC protein will eliminate the non-immunogenic intervening sequences. Collectively, the mvPC design demonstrates our goal to induce an effective immune response using a minimal well-defined antigen.

## Supplementary information

Supplementary Dataset 1.

Supplementary Dataset 2.

Supplementary Dataset 3.

Supplementary Dataset 4.
